# Delving into the role of reward and punishment sensitivity in anorexia nervosa: Punishment responsivity as the only predictor of eating disorder symptom persistence in adolescents

**DOI:** 10.1002/erv.3138

**Published:** 2024-10-01

**Authors:** Nienke C. Jonker, Klaske A. Glashouwer, Peter J. de Jong

**Affiliations:** ^1^ Department of Clinical Psychology and Experimental Psychopathology University of Groningen Groningen The Netherlands; ^2^ Accare Child and Adolescent Psychiatry Department of Eating Disorders Groningen The Netherlands

**Keywords:** adolescents, anorexia nervosa, eating disorders, motivation, punishment sensitivity, responsivity, reward sensitivity

## Abstract

**Objective:**

This study differentiated between self‐reported punishment responsivity (PR) and motivation to avoid punishment (MP) and examined their relationship with anorexia nervosa (AN) and its course in a combined cross‐sectional and longitudinal approach. We explored whether inconsistent findings regarding reward sensitivity may be explained by previous research not differentiating between reward responsivity (RR) and motivation to approach reward (MR).

**Method:**

Participants were 69 adolescents with AN and 69 adolescents without AN matched on age, sex and educational level. Eating disorder (ED) symptom severity, PR, MP, RR, and MR were assessed at the start of treatment and 1 year later.

**Results:**

Only PR was higher in patients with AN than in the comparison group. Both PR and MP decreased over the course of 1 year, however, only the reduction in PR was related to the reduction in ED symptoms. Lastly, only higher baseline PR was independently related to less improvement in ED symptoms over the course of 1 year.

**Conclusion:**

There was no support for the involvement of RS or its specific dimensions in AN. Most critical, the findings suggest that specifically the PR dimension of punishment sensitivity is related to the persistence of AN and could be an important target for treatment.

## Key points

- Adolescents with AN showed higher punishment responsivity than a comparison group without an eating disorder.
- Punishment responsivity was also the only factor that was independently related to the course of AN.
- This points to punishment responsivity as a promising target for treatment.

## 1 INTRODUCTION

Anorexia nervosa (AN) is a severe mental disorder marked by a striking ability to restrict food intake despite individuals with AN often being (severely) underweight (American Psychiatric Association, 2013). Established therapies for AN exhibit limited effectiveness, and relapse following successful treatment is a common occurrence (Brockmeyer et al., 2017; Khalsa et al., 2017). Punishment sensitivity (PS)—that is, sensitivity to negative or undesirable (punishing) stimuli—is identified as an important factor potentially contributing to the persistence of AN (e.g. Abber et al., 2024; Glashouwer et al., 2014; Jappe et al., 2011; Jonker et al., 2020). In line with this, high PS at the beginning of treatment was related to less improvement in eating disorder (ED) symptoms over the course of 1 year. Furthermore, the improvement in ED symptoms correlated with a concurrent reduction in PS. This points not only to the malleability of PS, but also to its promise as a target for treatment (Jonker, Glashouwer, & Jong, 2022).

PS can be subdivided in the response towards punishing stimuli called punishment responsivity (PR) and the motivation to avoid punishing stimuli (MP) (Jonker, Timmerman, & Jong, 2022). However, studies regarding AN used questionnaires that do not differentiate between PR and MP (Carver & White, 1994; Torrubia et al., 2001). Distinguishing between PR (e.g., extreme disappointment after weight gain) and MP (e.g., working very hard to avoid weight gain) could offer insight into which aspect of PS should be specifically addressed in treatment. Therefore, this study used the reward and punishment responsivity and motivation questionnaire (RPRMQ; Jonker, Timmerman, & Jong, 2022) to examine to what extent PR and MP are independently related to AN.

Reward sensitivity (RS) has been proposed as another potential factor in the persistence of AN, yet empirical research showed inconsistent findings. Studies reported lowered RS, heightened RS, or no differences in RS between individuals with versus without AN (Claes et al., 2006; Glashouwer et al., 2014; Jappe et al., 2011; Matton et al., 2015). RS can be subdivided in the response towards rewarding stimuli called reward responsivity (RR) and the motivation to approach rewarding stimuli (MR). However, thus far studies used global measures of RS. We will therefore explore whether inconsistent findings can be explained by RR and MR being differentially related to AN.

This study examined: whether (a) adolescents with AN differ in PR, MP, RR, and MR from individuals without AN, (b) PR, MP, RR, and MR change between start of treatment and 1 year later, (c) changes in PR, MP, RR, and MR are differentially related to changes in ED symptoms over the course of 1 year, and (d) the strengths of PR, MP, RR, and MR at the start of treatment are differentially related to change in ED symptoms over the course of 1 year.

## 2 METHOD

### 2.1 Participants

Participants were 69 adolescents (67 females, AgeMean = 15.55, SD = 1.70^1^) referred for inpatient or outpatient treatment to the Accare department of EDs in the Northern part of the Netherlands^2^. ED symptoms were assessed with the child version of the Dutch eating disorder examination (EDE) interview (Bryant‐Waugh et al., 1996; Decaluwé & Braet, 1999). Patients fulfilled DSM‐5 criteria for AN restrictive type (*n* = 39), AN binge purge (AN‐BP) type (*n* = 10), atypical AN restrictive type (*n* = 11), or atypical AN‐BP (*n* = 9). Patients reported that this was either their first (*n* = 62) or second AN episode (*n* = 7). 1 year after baseline, 62 participants (90%) completed the EDE, and 60 participants completed all follow‐up measures (87%). At follow‐up, 34 patients were still in treatment, 27 were not, and one patient did not provide this information.

For every patient an individually matched participant with a healthy weight was selected based on sex, age, and educational level resulting in a comparison group of 69 adolescents (67 females, Age_Mean_ = 15.48, SD = 1.82).

## 3 MATERIALS

### 3.1 BMI

Because BMI changes substantially with age, age and gender adjusted BMI was calculated ([actual BMI/median BMI for age and gender] × 100) (Cole et al., 2000).

### 3.2 ED symptoms

ED symptom severity was assessed with the ED Examination questionnaire (EDE‐Q; Fairburn & Beglin, 2008). Adaptations were made to make the language appropriate for adolescents (cf. Jansen et al., 2007). Items are scored on a seven‐point Likert scale and the average score of the 22 items was used to index ED symptom severity (Aardoom et al., 2012). Internal consistencies were excellent at baseline (for the patient and the comparison group Cronbach's alpha were 0.95 and 0.93, respectively), and at follow‐up (Cronbach's alpha = 0.97). In patients with AN, ED symptom severity was also indexed with the average score of the four subscales of the EDE‐interview (Bryant‐Waugh et al., 1996; Decaluwé & Braet, 1999). Internal consistencies were excellent at baseline and follow‐up (Cronbach's alpha = 0.89 and 0.94).

### 3.3 Punishment and reward sensitivity

Punishment and reward sensitivity were indexed with the reward and punishment responsivity and motivation questionnaire (RPRM‐Q; Jonker, Timmerman, & Jong, 2022). This questionnaire is a stimulus independent measure with 18 items indexing punishment responsivity (PR; five items, e.g., “I feel lousy after doing something wrong”), motivation to avoid punishment (MP; four items, e.g., “I avoid things that might have a negative outcome”), reward responsivity (RR; four items, e.g., “Obtaining rewards affects me strongly”), and motivation to approach reward (MR; five items, e.g., “I work hard for things that are potentially rewarding for me”). Items are answered on a five‐point Likert scale ranging from “this does not apply to me at all” to “this applies to me completely”. Internal consistencies during baseline were questionable to good in the comparison group (Cronbach's alpha = 0.89, 0.73, 0.60, and 0.70, respectively) and the patient group (Cronbach's alpha = 0.87, 0.67, 0.59, and 0.81, respectively). Internal consistencies during follow‐up in the patient group were acceptable to good (Cronbach's alpha = 0.89, 0.72, 0.81, and 0.87, respectively).

## 4 PROCEDURE

This study reports on data of a larger project on reward and punishment sensitivity in EDs which also examined punishment sensitivity with questionnaires that do not differentiate between PR and MP (Jonker et al., 2020, 2022), and was approved by the medical ethical committee of the University Medical Centre Groningen, the Netherlands (NL.51694042.14).

*Baseline:* Participants and their parents (when <18 years) signed informed consent. Baseline assessment took place at the treatment centre after intake (median 53 days after intake). For most participants this means that baseline assessment took place at the start of treatment or up to 4 weeks after. Some patients participated later for reasons such as hospital admission. The EDE‐interview was performed during intake and permission was asked to use this information for the current study. Medication use was derived from the patient files, and 11 patients were known to use medication. These were Olanzapine (*n* = 3), Fluoxetine (*n* = 4), Concerta (*n* = 1), Fluvoxamine (*n* = 1), Melatonin (*n* = 1), Sertraline (*n* = 1), Oxazepam (*n* = 1), and Thyrax (*n* = 1). Participants of the comparison group were recruited and tested at schools. During recruitment we specified that we were looking for adolescents without eating problems and with a healthy weight. During baseline, participants completed the RPRM‐Q, and EDE‐Q. Last, height and weight were measured.

*Treatment:* Treatment for AN provided at Accare is individually tailored. Participants (*n* = 60) reported to have received various therapy components, such as CBT‐Enhanced (Fairburn & Beglin, 2008; *n* = 50), diet management and exposure (*n* = 46), consultations with a dietician (*n* = 45), intensive family treatment (*n* = 34), and psychomotor therapy (*n* = 21).

*Follow‐up:* Follow‐up assessment took place 1 year after baseline (median 373 days) following the same procedure. Participants completed the RPRMQ and the EDE‐Q. Then, the EDE‐interview was administered and height and weight were measured.

## 5 RESULTS

### 5.1 Descriptives

Table 1 provides means and standard deviations of the variables during baseline for both groups and during follow‐up for patients with AN. Between baseline and follow‐up, BMI significantly increased and ED symptoms significantly decreased. Descriptives per AN subtype can be found in Supplement 1 in Supporting Information S1.

**TABLE 1:** Group characteristics.

	Comparison group (*n* = 69)		Patients with AN				Independent samples *t*‐test			Paired samples *t*‐test		
			Baseline (*n* = 69)		Follow‐up (*n* = 62)							
	Mean	SD	Mean	SD	Mean	SD	*t*	*d*	BF_10_	*t*	*d*	BF_10_
BMI	102.87	9.62	84.69	12.16	95.23^a^	14.91	−9.27**	1.58	>100	7.50**	0.96	>100
EDE^1^	^‐^	‐	3.74	1.10	1.81	1.51	‐	‐	‐	−9.49**	1.23	>100
EDE‐Q	1.30	1.10	4.16	1.11	2.57	1.58	15.17**	2.58	>100	−8.09**	1.05	>100
PR	3.49	0.97	4.10	0.77	3.87	0.96	4.12**	0.70	>100	−2.41*	0.31	3.98
MP	3.33	0.80	3.52	0.78	3.30	0.82	1.41	0.24	0.82	−2.61**	0.34	6.18
RR	4.05	0.55	3.86	0.64	3.75	0.88	−1.90	0.32	0.94	−1.32	0.17	1.32
MR	3.57	0.61	3.72	0.84	3.48	1.00	1.21	0.21	0.35	−2.06^a^	0.27	2.06

*Note*: ^a^ = *p* < 0.05, * = *p* < 0.01, **p < 0.001, ^1^ EDE was measured during intake. ^a^ BMI was available for 61 participants, EDE, EDE‐Q and RPRM‐Q for 60 participants at follow‐up. BF_10_ = Bayes factor quantifying the evidence in favour of the alternative hypotheses. BF_10_ between 3 and 10 is moderate evidence, and BF_10_ > 100 is very strong evidence that the data are more likely under the alternative hypothesis. BMI = Adjusted Body Mass Index. Adjusted BMI scores smaller than 85% are considered underweight and between 85% and 120% as normal weight (Winckel & Mil, 2001), EDE = Eating Disorder Examination interview, EDE‐Q = Total score on the Eating Disorder Examination Questionnaire, PR = Punishment Responsivity, MP = Motivation to avoid Punishment, RR = Reward Responsivity, MR = Motivation to approach Reward.

### 5.2 Do patients with AN differ in PR, MP, RR, and MR from comparisons without AN?

One‐sided independent samples *t‐*tests showed that PR, but not MP^3^ was significantly higher in patients with AN than in the comparison group (Table 1). Two‐sided independent samples *t‐*tests showed no differences for RR and MR. A post hoc exploration of the relationship between eating disorder symptoms, PR and MP and symptoms of anxiety and depression can be found in Supplement 2 in Supporting Information S1.

### 5.3 Do PR, MP, RR, and MR change between start of treatment and 1 year later?

Paired samples *t*‐test showed that PR, MP, and MR, but not RR, significantly reduced between baseline and follow‐up (Table 1).

### 5.4 Are changes in PR, MP, RR, and MR differentially related to change in ED symptoms?

Correlations between variables can be found in Table S5 (Supplement 3). Linear regression analyses with change in ED symptoms (EDE (model1) and EDE‐Q (model2)) as outcome measure and baseline ED symptoms as predictor showed that a greater reduction in PR was related to a greater decrease in ED symptoms (Table 2, step 2). Change in MP, RR, and MR did not independently predict change in ED symptoms.

**TABLE 2:** Regression models of change in eating disorder symptoms.

Dependent variable	Step	Independent	β	T	*P*_*T*_	Adj‐R^2^	F_change_	*P*_*F*_
Change in EDE (*n* = 57)	1	Baseline EDE	−0.39	4.0	0.003	0.14	9.76	0.003
	2	Change in PR	0.36	2.67	0.010	0.31	4.36	0.004
		Change in MP	0.13	0.83	0.410			
		Change in RR	−0.18	1.27	0.210			
		Change in MR	0.17	1.13	0.263			
	3	Baseline PR	0.39	2.12	0.039	0.37	2.24	0.079
		Baseline MP	−0.15	0.74	0.462			
		Baseline RR	−0.16	1.20	0.237			
		Baseline MR	0.06	0.39	0.697			
Change in EDE‐Q (*n* = 59)	1	Baseline EDE‐Q	−0.26	2.08	0.042	0.05	4.03	0.050
	2	Change in PR	0.37	2.67	0.010	0.23	4.26	0.005
		Change in MP	0.13	0.81	0.424			
		Change in RR	−0.04	0.27	0.786			
		Change in MR	0.11	0.72	0.477			
	3	Baseline PR	0.56	3.24	0.002	0.35	3.39	0.016
		Baseline MP	−0.40	1.93	0.060			
		Baseline RR	−0.10	0.67	0.506			
		Baseline MR	0.05	0.35	0.729			

*Note*: The power of these tests was at least 75% to find a medium effect. Change is calculated as follow‐up minus baseline. Thus, a lower change score means a stronger improvement in symptoms.

### 5.5 Are PR, MP, RR, and MR at the start of treatment differentially related to change in ED symptoms?

Adding baseline PR, MP, RR, and MR as predictor to the linear regression analyses (Table 2, step 3) showed that higher PR at baseline was related to less reduction in ED symptoms. MP, RR, and MR did not show an independent relationship with ED symptom change.^4^

## 6 DISCUSSION

The main outcomes are as follows: (a) Only PR, but not MP, RR, and MR, was higher in patients with AN than in the comparison group, (b) PR, MP, and MR, but not RR, significantly reduced over the course of 1 year, (c) a greater reduction in PR was the only independent predictor of a decrease in ED symptoms, (d) only higher baseline PR was independently related to less improvement in ED symptoms over the course of 1 year.

In line with previous studies that consistently showed high PS in patients with AN (Glashouwer et al., 2014; Jappe et al., 2011; Jonker et al., 2020; Matton et al., 2015), patients with AN showed higher PR than adolescents without an ED. A similar difference was not found for MP. In addition, although both PR and MP reduced over the course of 1 year, only change in PR was independently related to a change in ED symptoms. Furthermore, only PR at the start of treatment‐not MP‐was independently related to the reduction in ED symptoms over time. The previously reported finding that the change in general PS was related to a change in ED symptoms, and that general PS at the start of treatment was related to the reduction in ED symptoms (Jonker, Timmerman, & Jong, 2022) may thus be attributed to the responsivity dimension of PS. Together these findings suggest that specifically PR is involved in the persistence of AN and could be a promising target for treatment. Since higher PR is related to experiencing more negative emotions (Gray & McNaughton, 2000), one option could be to address emotion regulation skills to help patients cope with their strong response to punishing stimuli/experiences.

Post hoc analyses showed that change in PR and baseline PR were no longer significant predictors for the change in eating disorder symptoms after change in anxiety and change in depression were included in the model. Thus, although baseline and change in PR were related to improvement of patients with AN, it seems that this pattern of findings may be explained by the typical cooccurrence of AN and internalising symptoms. Interventions aimed at reducing PR might therefore be most effective in AN‐patients with relatively severe comorbid internalising symptoms.

In line with most evidence thus far showing no clear role for RS in AN, the current study showed no differences in RR and MR between adolescents with versus without AN. Furthermore, the change in RR and MR, and baseline RR and MR were not independently related to changes in ED symptoms over time. Thus, these findings suggest that differentiating between RR and MR does not help explain inconsistent findings of prior studies.

Strengths of the study are the large sample of adolescents with AN, low drop‐out rates, a longitudinal approach, and the assessment of ED symptoms with a diagnostic interview as well as self‐report. Some limitations should be kept in mind when interpreting these results. First, only adolescents were included in this study. Future studies should examine whether the findings also generalise to adult populations. Second, the RPRM‐Q is a new questionnaire and test‐retest reliability is still to be determined. Nevertheless, factor analyses have shown good fit of the items, and correlations between the subscales of the RPRM‐Q and the subscales of the more often used behavioural inhibition scale/behavioural activation scale (BIS/BAS; Carver & White, 1994) as well as the sensitivity to punishment and sensitivity to reward questionnaire (SPSRQ; Torrubia et al., 2001) support the validity (reliability) of the factors (Jonker, Timmerman, & Jong, 2022).

To conclude, this study showed that patients with AN do not differ from adolescents without an ED in motivation to avoid punishment but specifically report a higher punishment responsivity. Furthermore, specifically the change in PR, and the strength of PR at the start of treatment were related to a change in ED symptoms. These findings suggest that specifically punishment responsivity could be a relevant target for treatment.

## AUTHOR CONTRIBUTIONS

**Nienke C. Jonker:** Conceptualisation; data curation; formal analysis; funding acquisition; investigation; methodology; project administration; software; writing original draft; writing review and editing. **Klaske A. Glashouwer:** Conceptualisation; methodology; writing review and editing. **Peter J. de Jong:** Conceptualisation; funding acquisition; methodology; writing review and editing.

## CONFLICT OF INTEREST STATEMENT

The authors have no conflict of interest.

## FUNDING SOURCE

Data were collected as part of a project supported by a NWO research talent grant [406‐14‐091]. The laptops that were used in this study were funded by the Gratama foundation.

## Supporting information

Supplementary Material

## Data Availability

The data that support the findings of this study are available on request from the corresponding author. The data are not publicly available due to privacy or ethical restrictions.

## ENDNOTES

^1^
Only 8.7% (*n* = 6) of the participants were younger than 14 or older than 18 at baseline; so over 91% of all participants were aged 14–18.

^2^
Race and ethnicity were not assessed in light of national and EU laws stipulating that these special categories of (sensitive) data should not be processed unless absolutely necessary. However, in the northern parts of the Netherlands there is little ethnic diversity with people typically being from Dutch descent.

^3^
There was a significantly higher MP in adolescents with AN‐R (i.e., when excluding AN‐BP from analysis) than in the comparison group (*t*(117) = 2.37, *p* = 0.019, Cohen's *d* = 0.79). All other outcomes did not differ when AN‐BP were excluded from the analyses.

^4^
For both models (change in EDE and change in EDE‐Q) this finding was no longer significant when excluding patients with AN‐BP from the analyses.
