# Supplementary material for: Delving into the role of reward and punishment sensitivity in anorexia nervosa: Punishment responsivity as the only predictor of eating disorder symptom persistence in adolescents
Source: Eur Eat Disord Rev. 2024 Oct 1;33(1):174–80. doi: 10.1002/erv.3138 (PMC11617797; doi:10.1002/erv.3138)
Supplement: Supplementary file 1 — Supplementary Material [file ERV-33-174-s001.docx]

**Supplement 1** Descriptives per AN subtype

| Table S1. *Group characteristics of restrictive and binge purge subtypes.* | | | | | | | | |
| --- | --- | --- | --- | --- | --- | --- | --- | --- |
|  | **AN-R** | | | | **AN-BP** | | | |
|  | **Baseline**  **(*n* = 50)** | | **Follouw-up**  **(*n* = 45)** | | **Baseline (*n* = 19)** | | **Follow-up**  **(*n* = 15)** | |
|  | *Mean* | *SD* | *Mean* | *SD* | *Mean* | *SD* | *Mean* | *SD* |
| BMI | 82.72 | 10.86 | 94.05 | 13.78 | 89.96 | 14.06 | 98.53^3^ | 17.57 |
| EDE | 3.51^1^ | 1.11 | 1.67^2^ | 1.53 | 4.34 | 0.83 | 2.23^3^ | 1.40 |
| EDE-Q | 4.00 | 1.14 | 2.48 | 1.62 | 4.57 | 0.96 | 2.84 | 1.47 |
| PR | 4.28 | 0.69 | 3.97 | 0.94 | 3.63 | 0.80 | 3.55 | 0.95 |
| MP | 3.68 | 0.77 | 3.33 | 0.86 | 3.11 | 0.63 | 3.18 | 0.72 |
| RR | 3.96 | 0.53 | 3.74 | 0.93 | 3.58 | 0.82 | 3.78 | 0.74 |
| MR | 3.82 | 0.86 | 3.48 | 1.11 | 3.46 | 0.75 | 3.47 | 0.56 |
| *Note.* ^1^ *n* = 48, ^2^ *n* = 46, ^3^ *n* = 16. BMI = Adjusted Body Mass Index. Adjusted BMI scores smaller than 85% are considered underweight and between 85% and 120% as normal weight (Van Winckel & Van Mil, 2001), EDE = Eating Disorder Examination interview, EDE-Q = Total score on the Eating Disorder Examination Questionnaire, PR = Punishment Responsivity, MP = Motivation to avoid Punishment, RR = Reward Responsivity, MR = Motivation to approach Reward. | | | | | | | | |

**Supplement 2** Post-hoc analyses including anxiety and depression symptoms

Background

Punishment sensitivity has been suggested to be a risk factor for psychopathology in general and also for developing symptoms of anxiety and depression (e.g., Bijttebier, Beck, Claes, & Vandereycken, 2009; Gray, 1970). Furthermore, AN often co-occurs with symptoms of anxiety and depression. It is possible that the co-occurrence of symptoms can be partially explained by the increased sensitivity to punishment.

Symptoms of anxiety and depression

Symptoms of anxiety and depression were assessed with the Dutch version of the Revised Child Anxiety and Depression Scale (RCADS; Chorpita et al., 2000). The RCADS consists of 47 questions that can be answered on a 4-point scale ranging from never (0) to always (3). The depression subscale consists of 10 items. Anxiety was assessed by summing the items of the Social Phobia (9 items), Panic Disorder (9 items), Separation Anxiety (7 items) and Generalized Anxiety (6 items) subscales. In line with the DSM-5 categorization of anxiety disorders, the obsessive-compulsive subscale was not included in the anxiety score of the current study. The depression subscale showed acceptable to good internal consistencies (Cronbach’s alpha of .79 in patients with AN at baseline and .90 at follow-up, and of .87 in the comparison group at baseline). Internal consistencies of the anxiety scores were excellent (Cronbach’s alpha of .93 in patients with AN at baseline and .96 at follow-up and of .92 in the comparison group at baseline).

| Table S2. *Group characteristics* | | | | | | | | | | | | |
| --- | --- | --- | --- | --- | --- | --- | --- | --- | --- | --- | --- | --- |
|  | **Comparison group**  **(*n* = 69)** | | **Patients with AN** | | | | **Independent samples t-test** | | | **Paired samples t-test** | | |
|  |  |  | **Baseline (*n* = 69)** | | **Follow-up**  **(*n* = 62)** | |  |  |  |  |  |  |
|  | *Mean* | *SD* | *Mean* | *SD* | *Mean* | *SD* | *t* | *d* | *BF_10_* | *t* | *d* | *BF_10_* |
| Anxiety | 23.22 | 12.35 | 40.59 | 15.10 | 32.83 | 18.95 | 7.40** | 1.26 | >100 | 3.63** | 0.47 | >100 |
| Depression | 7.58 | 4.73 | 15.55 | 4.59 | 10.66 | 6.26 | 10.08** | 2.10 | >100 | 7.19** | 0.94 | >100 |
| *Note. ^a^ = p* < .05, * = *p* < .01, ** *p* < .001, ^1^ BF_10_ = Bayes factor quantifying the evidence in favor of the alternative hypotheses. BF_10_ between 3 and 10 is moderate evidence, and BF_10_ > 100 is very strong evidence that the data are more likely under the alternative hypothesis. Anxiety = RCADS anxiety, depression = RCADS depression. | | | | | | | | | | | | |

Cross-sectional analyses

Table S2 shows that patients with AN have more symptoms of anxiety and depression than the comparison group. To follow-up on the finding that adolescents with AN are more sensitive to punishment than adolescents without an eating disorder, we examined the relationship between sensitivity to punishment, eating disorder symptoms^[[1]](#footnote-1)^, and symptoms of anxiety and depression. PR was significantly related to eating disorder symptoms as measured with the EDE-Q (r = 0.30, p < .001), and significantly positively related to anxiety (r = 0.60, p < .001) and depression scores (r = 0.34, p < .001). MP was not significantly related to eating disorder symptoms as measured with the EDE-Q (r =-0.11, p = .203), or depression scores (r = 0.11, p = .218), but was significantly positively related to anxiety scores (r = 0.37, p < .001).

Additionally, we examined to what extent eating disorder symptoms as measured with the EDE-Q are independently related to punishment sensitivity, over and above symptoms of anxiety and depression. Therefore, hierarchical regression models were tested with PR and MP as dependent variables. All independent variables were centered before being entered into the models. In both models EDE-Q (PR: β = -.02, t = -0.20, p = .846; MP: β = -.02, t = -0.14, p = .886) showed no independent relationship with punishment sensitivity when symptoms of anxiety (PR: β = .84, t = 7.95, p < .001; MP: β = .71, t = 5.92, p < .001) and depression (PR: β = -.29, t = -2.25, p = .026; MP: β = -.43, t = -2.93, p = .886) were also included in the model. Thus, punishment responsivity and motivation to avoid punishment was not related to self-reported eating disorder symptoms over and above symptoms of anxiety and depression.

Longitudinal analyses

Table S2 shows that symptoms of anxiety and depression also significantly decrease over the course of one year. To examine to what extent the change in eating disorder symptoms is predicted by the change in punishment sensitivity and baseline punishment sensitivity over and above the change in symptoms of anxiety and depression we performed the regression analyses with the addition of change in anxiety and change in depression before adding the punishment and reward sensitivity scores (Table S3). The models show that the change in PR and baseline PR were no longer significant predictors of change in eating disorder symptoms.

| Table S3. Regression models of change in eating disorder symptoms (*N* = 59) | | | | | | | | |
| --- | --- | --- | --- | --- | --- | --- | --- | --- |
| **Dependent variable** | **Step** | **Independent** | **β** | **T** | ***P_T_*** | **Adj-R^2^** | **F_change_** | ***P_F_*** |
| Change in EDE | 1 | Baseline EDE | -.39 | 4.00 | .003 | .14 | 9.76 | .003 |
|  | 2 | Change in anxiety | .47 | 3.45 | <.001 | .40 | 13.17 | <.001 |
|  |  | Change in depression | .09 | 0.65 | .521 |  |  |  |
|  | 3 | Change in PR | .10 | 0.63 | .532 | .38 | 0.62 | .652 |
|  |  | Change in MP | .13 | 0.89 | .378 |  |  |  |
|  |  | Change in RR | -.09 | 0.66 | .514 |  |  |  |
|  |  | Change in MR | .05 | 0.30 | .764 |  |  |  |
|  | 4 | Baseline PR | -.10 | 0.73 | .469 | .46 | 2.68 | .044 |
|  |  | Baseline MP | -.03 | 0.24 | .816 |  |  |  |
|  |  | Baseline RR | .36 | 1.99 | .052 |  |  |  |
|  |  | Baseline MR | .03 | 0.17 | .869 |  |  |  |
| Change in EDE-Q | 1 | Baseline EDE-Q | -.26 | 2.08 | .042 | .05 | 4.03 | .050 |
|  | 2 | Change in anxiety | .42 | 3.10 | .003 | .40 | 17.24 | <.001 |
|  |  | Change in depression | .24 | 1.74 | .087 |  |  |  |
|  | 3 | Change in PR | .00 | 0.02 | .982 | .38 | 0.60 | .663 |
|  |  | Change in MP | .17 | 1.17 | .247 |  |  |  |
|  |  | Change in RR | .08 | 0.59 | .559 |  |  |  |
|  |  | Change in MR | -.11 | 0.69 | .492 |  |  |  |
|  | 4 | Baseline PR | -.04 | 0.32 | .750 | .45 | 2.80 | .036 |
|  |  | Baseline MP | -.02 | 0.11 | .914 |  |  |  |
|  |  | Baseline RR | .51 | 3.01 | .004 |  |  |  |
|  |  | Baseline MR | -.30 | 1.51 | .139 |  |  |  |
| *Note.* Change is calculated as follow-up minus baseline. Thus, a lower change score means a stronger improvement in symptoms. | | | | | | | | |

We additionally examined to what extent the change in anxiety and depression is predicted by the change in punishment and reward sensitivity and baseline punishment and reward sensitivity over and above the change in eating disorder symptoms. Table S4 shows that a greater reduction in PR and MR was related to a greater decrease in symptoms of both anxiety and depression over and above the change in ED symptoms. Furthermore, higher PR at baseline was related to less reduction in symptoms of depression.

| Table S4. Regression models of change in anxiety symptoms (*N* = 59) | | | | | | | | |
| --- | --- | --- | --- | --- | --- | --- | --- | --- |
| **Dependent variable** | **Step** | **Independent** | **β** | **T** | ***P_T_*** | **Adj-R^2^** | **F_change_** | ***P_F_*** |
| Change in Anxiety | 1 | Baseline anxiety | -.27 | 2.10 | .040 | .06 | 4.42 | .040 |
|  | 2 | Change in EDE | .58 | 5.40 | <.001 | .37 | 17.93 | <.001 |
|  | 3 | Change in PR | .51 | 4.73 | <.001 | .58 | 14.04 | <.001 |
|  |  | Change in MP | -.09 | 0.78 | .442 |  |  |  |
|  |  | Change in RR | -.13 | 1.15 | .256 |  |  |  |
|  |  | Change in MR | .26 | 2.14 | .037 |  |  |  |
|  | 4 | Baseline PR | .20 | 1.28 | .206 | .60 | 9.47 | <.001 |
|  |  | Baseline MP | -.27 | 1.56 | .125 |  |  |  |
|  |  | Baseline RR | -.13 | 1.18 | .243 |  |  |  |
|  |  | Baseline MR | .10 | 0.88 | .384 |  |  |  |
| Change in Depression | 1 | Baseline depression | -.21 | 1.61 | .113 | .03 | 2.60 | .113 |
|  | 2 | Change in EDE | .46 | 3.78 | <.001 | .22 | 8.76 | <.001 |
|  | 3 | Change in PR | .54 | 4.49 | <.001 | .48 | 9.51 | <.001 |
|  |  | Change in MP | -.23 | 1.74 | .089 |  |  |  |
|  |  | Change in RR | -.21 | 1.66 | .104 |  |  |  |
|  |  | Change in MR | .46 | 3.41 | .001 |  |  |  |
|  | 4 | Baseline PR | .36 | 2.24 | .030 | .55 | 7.78 | <.001 |
|  |  | Baseline MP | -.02 | 0.08 | .936 |  |  |  |
|  |  | Baseline RR | -.08 | 0.65 | .517 |  |  |  |
|  |  | Baseline MR | -.05 | 0.42 | .678 |  |  |  |
| *Note.* Change is calculated as follow-up minus baseline. Thus, a lower change score means a stronger improvement in symptoms. | | | | | | | | |

Discussion

In patients with AN, ED symptoms and symptoms of anxiety and depression often co-occur. With these additional analyses we show that punishment sensitivity was no longer a significant predictor of eating disorder symptoms after anxiety and depression (in the case of PR) and depression (in the case of MP) symptoms were included in the analyses. In other words, heightened PS scores in patients with AN might, at least partly, reflect heightened symptoms of anxiety and depression.

Furthermore, change in PR and baseline PR no longer were significant predictors for the change in eating disorder symptoms over time after change in anxiety and change in depression were already included in the model. However, the other way around, change in PR was a significant predictor for the change in symptoms of anxiety and depression over time after change in ED symptoms was already included in the model. Thus, also the relationship between punishment sensitivity and the change in eating disorder symptoms over time might reflect the relationship between the change in punishment sensitivity and the change of symptoms of anxiety and depression over time.

All in all, it seems that there is a complex interrelationship between ED symptoms, symptoms of anxiety and depression, and punishment sensitivity. Although the findings showed that the change in PR and baseline PR are related to improvement of patients with AN, it seems that this pattern of findings may be explained by the typical cooccurrence of AN and internalizing symptoms. One potential implication of the findings could be that an intervention aimed at reducing PR might be most effective in patients with AN with relatively severe symptoms of anxiety and depression.

**References**

Bijttebier, P., Beck, I., Claes, L., & Vandereycken, W. (2009). Gray’s Reinforcement Sensitivity Theory as a framework for research on personality-psychopathology associations. *Clinical Psychology Review, 29,* 421–430. http://doi.org/10.1016/j.cpr.2009.04.002

Chorpita, B. F., Yim, L., Moffitt, C., Umemoto, L. A., & Francis, S. E. (2000). Assessment of symptoms of DSM-IV anxiety and depression in children: A revised child anxiety and depression scale. *Behaviour Research and Therapy, 38,* 835–855. http://doi.org/10.1016/S0005-7967(99)00130-8

Gray, J. A. (1970). The psychophysiological basis of introversion-extraversion. *Behaviour Research and Therapy, 8,* 249–266. http://doi.org/10.1016/0005-7967(70)90069-0

**Supplement 3** Correlations between variables included in the regression analyses.

| Table S5. *Correlations between variables.* | | | | | | | | | |
| --- | --- | --- | --- | --- | --- | --- | --- | --- | --- |
|  | 1. | 2. | 3. | 4. | 5. | 6. | 7. | 8. | 9. |
| 1. Change EDE^1^ | - | - | - | - | - | - | - | - | - |
| 2. Change EDE-Q^2^ | .71*** | .67*** | - | - | - | - | - | - | - |
| 3. Change PR | .46*** | .45*** | - | - | - | - | - | - | - |
| 4. Change PM | .36** | .37** | .54*** | - | - | - | - | - | - |
| 5. Change RR | .06 | .15 | .18 | .40** | - | - | - | - | - |
| 6. Change RM | .22 | .24 | .17 | .51*** | .62*** | - | - | - | - |
| 7. PR baseline | .08 | .09 | -.29* | -.23 | .12 | -.02 | - | - | - |
| 8. PM baseline | -.05 | -.18 | -.28* | -.45*** | -.08 | -.14 | .64*** | - | - |
| 9. RR baseline | -.22 | -.27* | -.28* | -.20 | -.16 | -.03 | .20* | .40*** | - |
| 10. RM baseline | -.14 | -.20 | -.19 | -.24 | -.05 | -.39** | .18* | .36*** | .41*** |
| *Note.* ^1^ Controlling for baseline EDE, ^2^ Controlling for baseline EDE-Q, ***p < .001, ** p < .01, * p < .05. | | | | | | | | | |

1. Since the EDE was only available for the patient group and not the comparison group, EDE scores were left out of these analyses. [↑](#footnote-ref-1)
